# Gene flow, kinship, and source population assignment in *Chrysomya* blow flies

**DOI:** 10.1038/s41598-026-66124-w

**Published:** 2026-08-29

**Authors:** Abeer M. Salem, Christine J. Picard, Ioannis Eleftherianos, Eman E. Zaher, Rasha K. Al-Akeel, Hathal M. Al Dhafer

**Affiliations:** 1 Department of Biotechnology, Faculty of Science, Cairo University, P.O. Box 12613, Giza, Egypt; 2 Department of Biology, Indiana University Indianapolis, 723 W Michigan Street, Indianapolis, IN 46202 USA; 3 School of Biological Sciences, Queen’s University Belfast, Belfast, UK; 4 Department of Zoology, Faculty of Science, Zagazig University, Zagazig, Egypt; 5 Department of Zoology, Faculty of Science, King Saud University, 11451 Riyadh, Saudi Arabia; 6 King Saud University Museum of Arthropods (KSMA), Plant Protection Department, College of Food and Agriculture Sciences, King Saud University, P.O. Box 2460, 11451 Riyadh, Saudi Arabia

**Keywords:** AFLP, *Chrysomya albiceps*, *Chrysomya marginalis*, Forensic entomology, Population genetic structure, Ecology, Ecology, Evolution, Genetics, Zoology

## Abstract

Blow flies of the genus *Chrysomya* are among the earliest colonizers of human remains and are important forensic indicators in Africa and the Middle East. However, the population genetic structure of Old World *Chrysomya* species in North Africa remains poorly understood. We conducted the first amplified fragment length polymorphism (AFLP)-based population genetic analysis of *Chrysomya albiceps* and *Chrysomya marginalis* in Egypt to assess geographic and temporal structure and to evaluate their forensic utility for population assignment. Adult flies were collected from multiple localities in Egypt and across consecutive years, and AFLP profiles were generated using four primer combinations. All individuals yielded unique multilocus genotypes. In *C. albiceps*, most genetic variation occurred within populations (93%), with only weak but significant among-population differentiation (Fst = 0.045), no isolation by distance, and no geographically coherent clustering, indicating near-panmixia at the regional scale. In *C. marginalis*, spatial differentiation was negligible, whereas temporal comparison of samples from Giza revealed modest year-to-year structuring. Within-sample relatedness was consistently positive in both species, suggesting nonrandom kin associations within collection events. Although simulation-based assignment success was high, empirical reallocation performance was substantially lower, indicating that assignment accuracy depends strongly on the level of genetic differentiation and may not yet be robust for routine forensic inference. This apparent paradox reflects fine-scale structuring driven by kin aggregation within sampling events, despite overall high gene flow at the regional scale.

**Supplementary Information:**

The online version contains supplementary material available at https://doi.org/10.1038/s41598-026-66124-w.

## Introduction

Population genetics provides the framework for interpreting how migration, drift, and selection structure variation within and among insect populations, but in forensic contexts that framework has direct evidentiary consequences rather than purely theoretical ones^1,2^. Blow flies are especially informative because they arrive rapidly on remains and often do so in cohesive cohorts rather than as random representatives of a regional pool^3^. Consequently, the genetic profile of larvae or adults collected from carrion may reflect kin structure, local recruitment, and source-population history, all of which affect whether insect evidence can support postmortem interval estimation or inference of corpse movement^4–7^. In this sense, the central question is not merely whether blow flies disperse widely, but whether dispersal erases enough structure to make individual samples unusable for forensic assignment^8^. Despite their forensic importance, the population genetic structure of Egyptian Old World *Chrysomya* species remains largely unexplored, particularly regarding the relative contributions of geographic separation, temporal turnover, and fine-scale kin structure to patterns of genetic variation. This knowledge gap limits the interpretation of forensic entomological evidence because blow flies are highly mobile insects that readily exploit ephemeral carrion resources, potentially promoting extensive gene flow while simultaneously generating localized cohorts of closely related individuals. Consequently, genetic differentiation observed among forensic samples may reflect true population subdivision, temporal recruitment dynamics, or the non-random collection of related flies rather than geographic isolation alone. Disentangling these processes is therefore essential for evaluating the reliability of population assignment and the use of blow fly genetic evidence in forensic investigations.

That question is complicated by the fact that developmental variation among blow-fly populations can arise from both environment and genotype. Work on *Lucilia sericata* has shown that developmental rate, pupal size, and minimum development time vary among geographically separated colonies and across temperatures, while other studies have emphasized that differences among datasets may reflect rearing conditions, strain effects, or both^9–12^. Forensic interpretation is therefore vulnerable to a mismatch between biological reality and the assumptions embedded in laboratory growth curves: a population-specific developmental signature can bias postmortem interval estimates, whereas environmental plasticity can obscure genuine genetic differentiation. The literature thus suggests that phenotypic variability alone is an imperfect proxy for population structure and that direct genetic assessment is required^13^. Recent empirical data on seasonal temperature variations and laboratory-derived development data for *Chrysomya rufifacies* further underscore the importance of accounting for cohort-specific developmental plasticity when interpreting forensic samples^14^.

Genome-wide neutral markers are therefore essential for distinguishing true population differentiation from environmentally driven phenotypic variation^15^. Amplified fragment length polymorphism (AFLP) remains a valuable approach for population genetic studies of non-model insects because it generates large numbers of polymorphic loci without prior genomic information and has been successfully applied to population assignment, kinship analysis, and forensic inference in blow flies and other taxa^4,5,16–19^. Its use in the present study also permits direct comparison with previous forensic investigations of *Chrysomya* and other calliphorid species that employed the same marker system^4,5,17^. Although high-resolution genomic approaches are now available, our objective was to investigate broad-scale population genetic structure, relatedness, and forensic assignment potential rather than fine-scale population genomic variation. Consequently, AFLP provides an appropriate and well-established genome-wide marker system that offers sufficient resolution to address these objectives while maintaining methodological continuity with previous forensic entomological studies^4,5,16–19^.

The present study was therefore designed to evaluate the reproducibility and resolution of an AFLP-based workflow in Egyptian *Chrysomya* spp., to determine whether genetic differentiation is better explained by spatial or temporal separation, and to test whether relatedness and assignment analyses provide a defensible basis for forensic source attribution. We hypothesized that broad geographic structure would be weak, but that samples collected from the same bait or narrow collection window would retain detectable kin structure and species-specific assignment signal.

## Materials and methods

### Specimen collection

Adult *Chrysomya albiceps* (Wiedemann) were collected from four geographically separated localities in Egypt between March and June 2013: Giza (n = 36; 30°00′N, 31°10′E; 27 May 2013), Minya (n = 9; 30°07′N, 30°33′E; 13 March 2013), Dayrout (n = 24; 27°34′N, 30°49′E; 4 June 2013), and North Sinai (n = 8; 31°02′N, 33°00′E; 2 May 2013), with a maximum inter-site distance of approximately 500 km (Table 1). At Minya and North Sinai, specimens were captured within a 30-min window using a sweeping net deployed at a fish-baited trap. At Giza and Dayrout, fish-baited traps were operated for 3 h. Because the initial Dayrout collection yielded an insufficient number of specimens for analysis, an additional fish-baited trap was established approximately 10 km from the primary sampling site to increase sample size within the same geographic area. Owing to their close spatial proximity and the absence of major geographic barriers, specimens from the two Dayrout sites were pooled and treated as a single population for subsequent genetic analyses. Adult *Chrysomya marginalis* (Wiedemann) were obtained in two collection campaigns. In 2013, 10 individuals were collected from two localities: Giza (n = 7; 30°00′N, 31°10′E; 27 May 2013) and Dayrout (n = 3; 27°34′N, 30°49′E; 4 June 2013), using fish-baited traps. In 2014, an additional 17 adults were collected from Giza (30°00′N, 31°10′E; 5 May 2014) by sweeping net at an open fish market over a 3-h collection period (Table S1). All specimens were immediately killed by immersion in 70% ethanol and stored at − 20°C until DNA extraction.

**Table 1:** *Chrysomya albiceps* collection sites, sample sizes, collection dates, geographic coordinates, percentage of polymorphic AFLP loci, and within-sample Nei’s gene diversity (Hj).

Collection site	Coordinates	Collection date	n (sex)	% Polymorphism*	Hj ± SE
Giza	30°00′N / 31°10′E	27 May 2013	36 (♀)	72.9	0.201 ± 0.005
Minya	30°07′N / 30°33′E	13 March 2013	9 (♀)	61.6	0.193 ± 0.006
Dayrout	27°34′N / 30°49′E	4 June 2013	24 (♀)	67.4	0.155 ± 0.007
North Sinai	31°02′N / 33°00′E	2 May 2013	8 (♀)	74.2	0.240 ± 0.007
Total	–	–	77	–	–

*Percentage of loci with minor allele frequency ≥ 5% of total AFLP loci scored.

### AFLP genotyping

AFLP profiling was performed on all individuals following the protocol of Picard and Wells^4^ with minor modifications. The complete workflow comprised four sequential steps: restriction digestion, adaptor ligation, two-stage PCR amplification, and capillary electrophoresis (CE). All oligonucleotide sequences are provided in Table S2.

#### DNA extraction

Genomic DNA was extracted from the head tissue of each fly to minimize contamination from eggs, ingested material, and gut-associated microorganisms. DNA extraction was performed using the DNeasy Blood & Tissue Kit (Qiagen, Valencia, CA, USA) following the manufacturer’s protocol, and purified DNA was stored at − 20 °C. DNA concentration and purity were assessed using a NanoDrop spectrophotometer (Thermo Fisher Scientific, Waltham, MA, USA), while DNA integrity was verified by electrophoresis on a 1% agarose gel. The remaining body tissues were preserved in ethanol at − 20 °C as voucher specimens at Indiana University–Purdue University Indianapolis.

#### Restriction digestion

Fifteen microlitres of extracted genomic DNA was subjected to simultaneous double-digestion in a 50-µL reaction containing 2 U *Pst*I, 2 U *Eco*RI, 5 µL of 10 × Buffer H, and 0.5 µL of 1 × bovine serum albumin (BSA); all enzymes and buffers were supplied by Promega Corporation (Madison, WI, USA). The reaction volume was adjusted to 50 µL with nuclease-free water (NFH_2_O). Digestion proceeded at 37°C for 3 h in a thermal cycler, followed by enzyme inactivation at 70°C for 15 min. Digested products were held at 4°C pending ligation.

#### Adaptor preparation and ligation

Double-stranded adaptors were prepared separately for each enzyme. For each adaptor pair, 3 µg of each single-stranded oligonucleotide (Table 2) was combined with 6 µL of 10 × Buffer H (Promega) and NFH_2_O to a total volume of 120 µL. Each solution was denatured at 65°C for 10 min and allowed to anneal by cooling to room temperature. Working stocks of all primers (25 pg/µL) and adaptors (3 µg/µL) were prepared in NFH_2_O and stored at − 20°C.

**Table 2:** AMOVA results for *Chrysomya albiceps* based on 542 AFLP loci and 999 permutations.

Source of variation	df	SS	MS	Est. Var	Φ statistic	%	P
Among populations	3	545.869	181.956	5.965	ΦPT = 0.069	7%	0.001
Within populations	73	5908.611	80.940	80.940	ΦPR	93%	–
Total	76	6454.481	–	86.905	–	100%	–

Ligation was performed by adding 1 µL of each double-stranded adaptor and 1 µL of 10 × DNA ligase buffer (Promega) directly to the 50-µL digestion product, followed by 1 U T4 DNA ligase (Promega) and NFH_2_O to a final volume of 62 µL. Ligation mixtures were incubated at room temperature for 3–4 h with gentle agitation applied at 1-h intervals.

To assess inter-replicate reproducibility of the AFLP workflow, 10 independent aliquots were prepared from the ligation product of a single representative individual. Each aliquot was processed through the complete pre-selective and selective amplification workflow alongside the study samples.

#### Pre-selective PCR

Pre-selective amplification was performed in a 20-µL reaction containing 2 µL of ligation product, 10 µL of 2 × PCR Master Mix (Promega), 1 µL of *Eco*RI + A primer (25 pg/µL), 1 µL of *Pst*I + A primer (25 pg/µL), and 6 µL NFH_2_O. Cycling conditions on a Mastercycler thermal cycler (Eppendorf Inc., Hamburg, Germany) were: initial denaturation at 94°C for 2 min; 26 cycles of 94°C for 1 min, 56°C for 1 min, and 72°C for 2 min; and a final extension at 72°C for 5 min. Pre-selective products were diluted 1:10 in NFH_2_O (i.e., 10 µL product added to 100 µL NFH_2_O) and 1 µL of the diluted product was used as template for selective PCR.

#### Selective PCR

Selective amplification was performed in four independent 20-µL reactions per individual, each using a different *Pst*I selective primer (*Pst*I + ACG, *Pst*I + AGT, *Pst*I + AAC, or *Pst*I + ATC; Table 2). Each reaction contained 1 µL of diluted pre-selective product, 10 µL of 2 × PCR Master Mix (Promega), 1.5 µL of FAM-labelled *Eco*RI selective primer (Applied Biosystems, Foster City, CA, USA), 1.5 µL of the respective *Pst*I selective primer (25 pg/µL), and 6 µL NFH_2_O. Cycling conditions comprised: initial denaturation at 94°C for 2 min; 10 touchdown cycles of 94°C for 30 s, annealing from 65°C to 56°C for 30 s (decreasing by 1°C per cycle), and 72°C for 2 min; 27 cycles of 94°C for 30 s, 56°C for 30 s, and 72°C for 2 min; and a final extension at 72°C for 10 min.

#### Capillary electrophoresis

For fragment sizing, 0.5 µL of each selective PCR product was combined with 9.7 µL of Hi-Di formamide and 0.3 µL of LIZ600 internal size standard (both Applied Biosystems), denatured at 95°C for 10 min, and immediately snap-chilled on ice. Capillary electrophoresis was performed on an Applied Biosystems 3500 Genetic Analyzer (Life Technologies Inc.) using default instrument parameters for AFLP fragment analysis.

### AFLP data scoring and locus filtering

Raw electropherograms were imported into GeneMarker v2.4 (SoftGenetics LLC, State College, PA, USA). Fragment detection was carried out using the default peak-calling settings with two modifications: (i) the fluorescence detection threshold for the FAM channel was set to 100 relative fluorescence units (RFU), and (ii) peak heights were normalised to the maximum signal intensity within each project. Only fragments in the size range 100–500 bp were retained to minimise size homoplasy^20^. Scored profiles were exported as binary presence/absence matrices for each primer combination.

Loci were filtered in Microsoft Excel (Microsoft Corp., Redmond, WA, USA) to retain only those with intermediate allele frequencies (0.05–0.95), thereby excluding rare and near-fixed AFLP fragments that contribute little to estimates of population differentiation and may introduce scoring noise and homoplasy bias^20–23^. Following filtering, 542 and 507 polymorphic loci were retained for *C. albiceps* and *C. marginalis*, respectively.

### Genetic diversity

Within-sample genetic diversity was characterised by two complementary metrics. First, the percentage of polymorphic loci (%P) was calculated per population using a 5% minor-allele frequency criterion. Second, Nei’s gene diversity (H_j_) was estimated for each population sample, and total population diversity (H_T_) across all samples, using AFLP-SURV v1.0^20^ according to the Lynch and Milligan^24^ correction for dominant markers.

### Population genetic structure

#### Analysis of molecular variance

The partitioning of AFLP genetic variance within and among populations was assessed by Analysis of Molecular Variance (AMOVA) implemented in GenAlEx 6.5^25,26^. Variance components were estimated at three hierarchical levels: within populations (Φ_PR_), among populations within regions (Φ_PT_), and among regions (Φ_RT_). The Φ statistic—an analogue of Wright’s F_st_ suitable for band-based dominant marker data—was calculated from squared Euclidean distances among AFLP profiles and does not rely on distributional assumptions that may underestimate genetic variability^27,28^. Statistical significance of all variance components and pairwise genetic distances was evaluated by 999 random permutations. The overall fixation index (F_st_) was estimated independently using AFLP-SURV, which calculates allele frequencies from dominant AFLP markers following the method of Lynch and Milligan^24^. Both ΦPT and F_st_ were used as complementary measures of population differentiation.

#### Isolation by distance

A Mantel test was performed in GenAlEx 6.5 to evaluate the correlation between matrices of pairwise genetic distance (Φ_PT_) and Euclidean geographic distance (km), calculated from GIS coordinates of all collection sites. The Pearson product-moment correlation coefficient (*R*) was used as the test statistic; its significance was assessed using SPSS (IBM Corp., Armonk, NY, USA).

#### Bayesian clustering analysis

Bayesian model-based clustering was performed in STRUCTURE v2.3.4^29^ to identify genetically distinct subpopulations within each dataset. AFLP loci were scored as binary markers, with fragment presence coded as "1", absence coded as "0", and missing data coded as "-9" in the STRUCTURE input file. Analyses assumed an admixture model with independent allele frequencies. The number of clusters (*K*) was explored from 1 to 9; for each value of *K*, 10 independent runs were performed with a burn-in of 10,000 iterations followed by 50,000 Markov chain Monte Carlo (MCMC) iterations, sufficient to stabilize likelihood estimates. Analyses were conducted on the full dataset and, to detect hierarchical substructure, on each geographic population individually^30^. The optimal *K* was determined using Structure Harvester v0.6.94^31^ by plotting the second-order rate of change in log-likelihood (Δ*K*) against *K*; the *K* value corresponding to the highest Δ*K* peak was considered the most probable number of genetic clusters.

#### Principal coordinate analysis

Principal coordinate analysis (PCoA) was performed in GenAlEx 6.5 using the matrix of pairwise Φ_PT_ genetic distances as input, to provide a geometry-free validation of STRUCTURE cluster assignments and to visualise the principal axes of genetic variation among populations.

### Within-sample relatedness

Pairwise relative relatedness coefficients (*r*) were estimated for all individuals within each population sample using SPAGeDi v1.3^32^. Coefficients were calculated according to Hardy^33^, using allele frequencies estimated across the full dataset as the reference. Because an empirical inbreeding coefficient was unavailable, a value of zero was assumed—a standard and analytically justifiable default, as Hardy^33^ demonstrated the estimator is robust to violations of this assumption, and Picard and Wells^5^ confirmed that inbreeding coefficients ranging from 0 to 1 exerted no statistically significant effect on mean *r* in comparable blow fly datasets. Within-sample means and standard deviations of pairwise *r* were compared with a negative control constructed by randomly selecting one individual per geographic region, thereby minimising kinship in the reference set.

### Population assignment analysis

Individual-level assignment tests were performed using AFLPOP v2.0^34^, which calculates log-likelihood statistics to assign each specimen to the source population in which its multilocus genotype has the highest expected frequency. Zero-frequency alleles were replaced by the correction factor 1/(sample size + 1) to account for unsampled alleles.

Assignment stringency was governed by the minimum likelihood difference (MLD) parameter: an individual was assigned to population *X* only if the log-likelihood of its genotype in *X* exceeded the log-likelihood in every other population by at least MLD units (i.e., the genotype was MLD-fold more probable in *X* than in any alternative). Higher MLD values reduce misallocation rates at the cost of increasing the proportion of unassigned individuals. The optimal MLD for each species was determined using the *Simulation: many iterations* procedure by evaluating MLD values from 0 to 15. For each candidate MLD, 1,000 random genotypes per population were simulated over 10 iterations, and the lowest MLD that minimized misallocation while maximizing the proportion of correctly assigned individuals without substantially increasing the proportion of unassigned individuals was selected. Two complementary procedures were then applied at the optimised MLD. The *Simulation* procedure quantified the theoretical upper bound on assignment success under ideal conditions: 1,000 random genotypes per population (10 iterations) were generated and allocated to their source populations, yielding a simulated assignment success rate. The *Re-allocation* procedure quantified empirical performance: each individual fly was iteratively excluded from its population’s dataset, population allele frequencies were recalculated from the remaining individuals, and the excluded fly was then assigned to the most likely population. The proportion of correctly assigned, misassigned, and unassigned individuals was recorded for each population. Assignment success was defined as the ratio of correctly allocated individuals to the total number of individuals subjected to assignment. The discrepancy between *Simulation* and *Re-allocation* success rates was used as a measure of the practical forensic utility of each species’ genetic profile for postmortem corpse relocation inference.

To facilitate comparison among closely related forensic blow fly species, the previously published AFLP dataset for *C. megacephala*, generated using the same laboratory protocols and analytical workflow, was reanalyzed alongside the newly generated datasets for *C. albiceps* and *C. marginalis*. Because the *C. megacephala* dataset has been described in detail elsewhere^15^, it is included here solely for comparative analyses.

## Results

### Chrysomya albiceps

#### Specimen collection and AFLP genotyping

A total of 77 adult female *C. albiceps* were collected from four geographically separated localities in Egypt between March and June 2013: Giza (n = 36), Minya (n = 9), Dayrout (n = 24), and North Sinai (n = 8) (Table 1). Giza and Dayrout were sampled over a 3-h trapping period using fish-baited traps, whereas specimens from Minya and North Sinai were collected within a 30-min interval using sweep nets at baited traps. Because the initial Dayrout collection yielded an insufficient number of specimens for population-level analyses, individuals from a second sampling site located < 10 km away were pooled with those from the primary site to increase sample size while representing the same local population. Four selective primer combinations generated 1,038 AFLP loci, of which 542 polymorphic loci were retained after quality filtering. All individuals possessed unique multilocus genotypes. The proportion of polymorphic loci ranged from 61.6% to 74.2%, and Nei’s gene diversity (Hj) ranged from 0.155 ± 0.007 to 0.240 ± 0.007 (Table 1).

#### Genetic and geographic structure

**Fig. 1:**
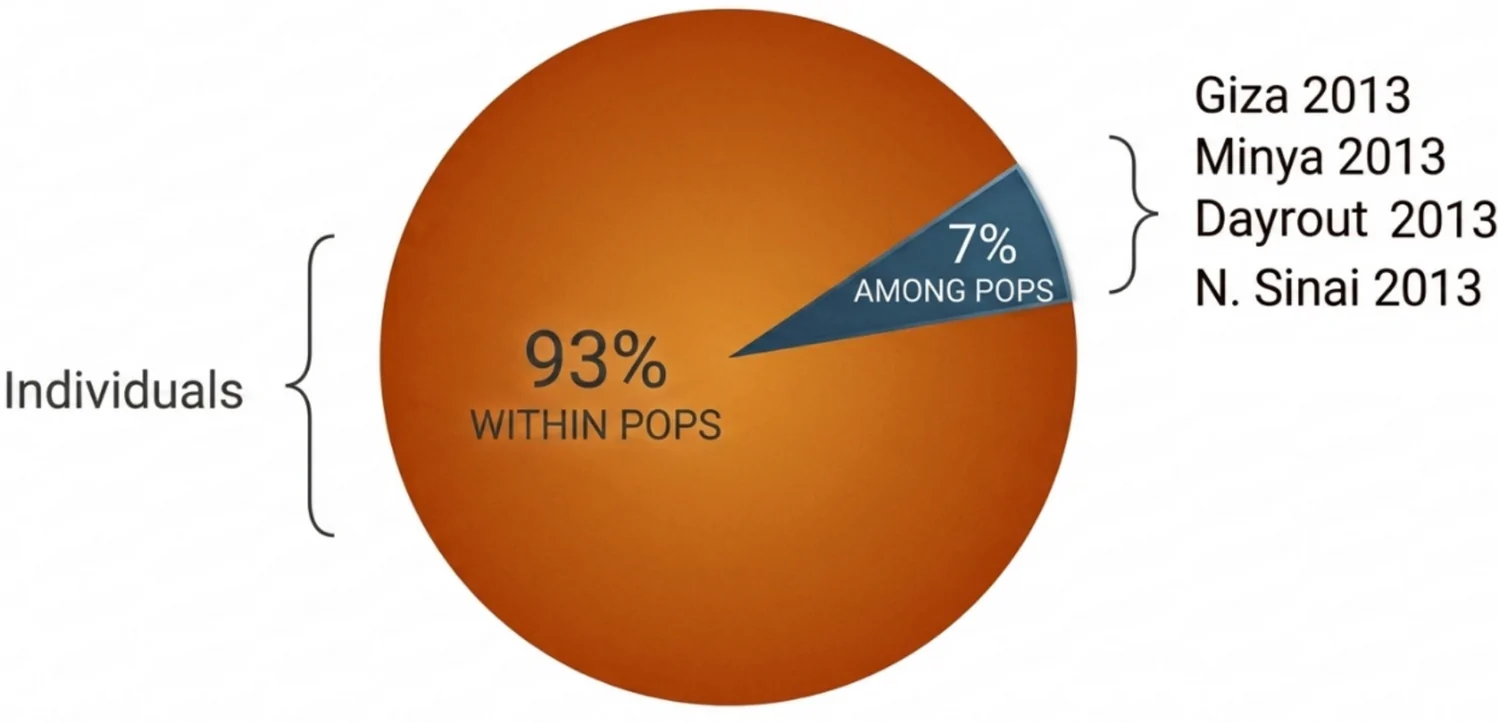
Analysis of molecular variance (AMOVA) showing the partitioning of genetic variation in *Chrysomya albiceps*. The pie chart illustrates that most genetic variation is distributed within populations (93%), with a smaller proportion attributable to among-population differentiation (7%), based on 542 AFLP loci.

Analysis of molecular variance (AMOVA) partitioned the total AFLP genetic variation predominantly within populations (Φ_PR_ = 93%, P = 0.001), with a small but statistically significant proportion residing among populations (Φ_PT_ = 7%, P = 0.001) (Table 2; Fig. 1). The pairwise Mantel test between Euclidean geographic distances and Φ_PT_-based genetic distances yielded a non-significant negative correlation (R^2^ = 0.15, P = 0.32; SSx = 60,384.408, SSy = 0.0181, SPxy =  − 12.879, Rxy =  − 0.388), indicating no evidence of isolation by distance (IBD) across the four *C. albiceps* populations (Table S1; Figure S3).

The fixation index F_st_ = 0.0453 ± 0.23 and total gene diversity H_T_ = 0.207 were both statistically significant (P = 0.001), confirming slight but detectable interpopulation genetic differentiation in the absence of a geographic correlation structure.

**Fig. 2:**
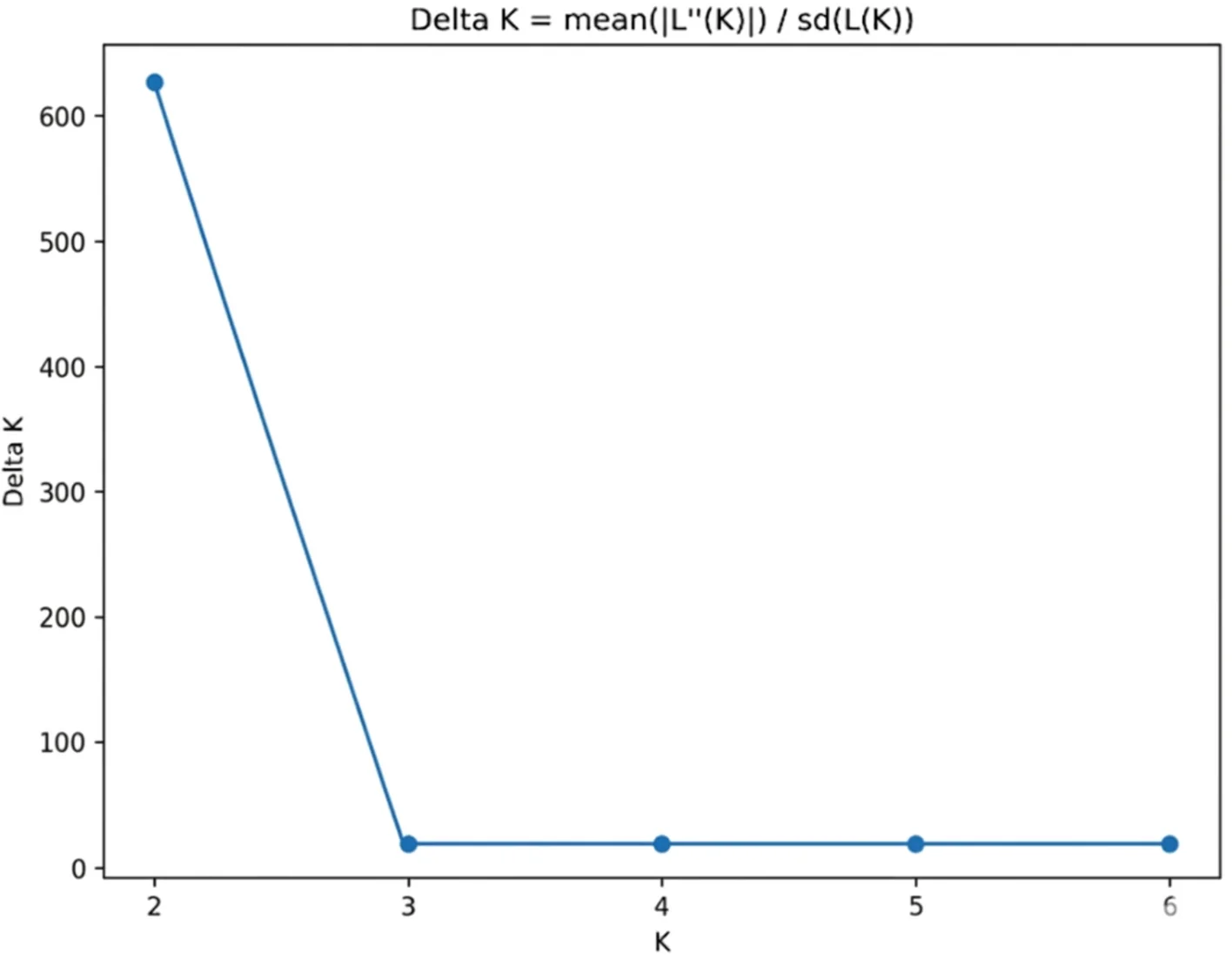
ΔK plot from STRUCTURE HARVESTER analysis of *Chrysomya albiceps* (K = 1–9), indicating maximum support at K = 2.

**Fig. 3:**
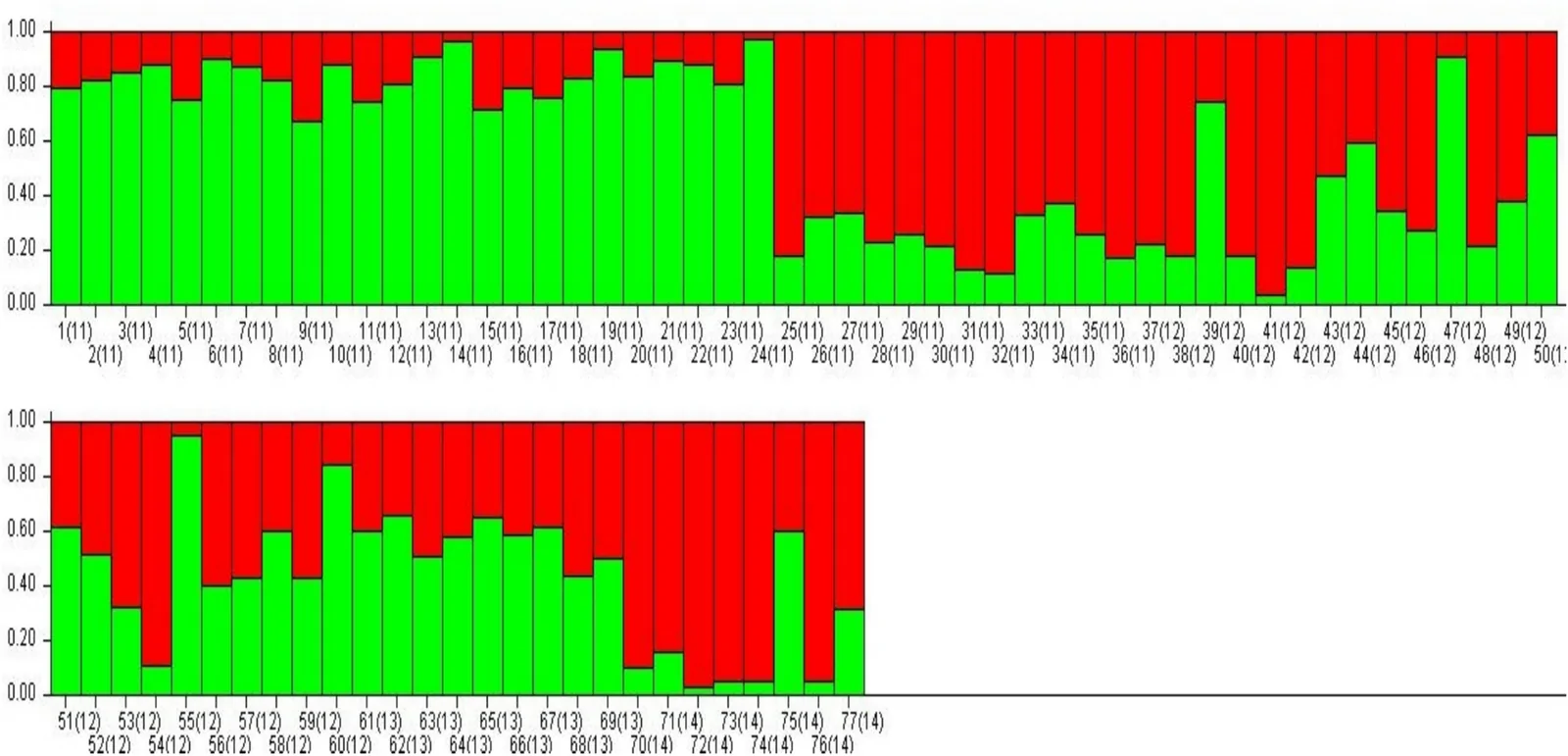
STRUCTURE bar plot for *Chrysomya albiceps*at K = 2. Each vertical bar represents one individual; colors denote estimated membership proportions to each of the two inferred clusters. Population codes: 11 = Giza, 12 = Dayrout, 13 = Minya, 14 = North Sinai.

STRUCTURE Bayesian clustering, performed for K = 1–9 with 10 independent runs (burn-in = 10,000; MCMC = 50,000 iterations), identified K = 2 as the most likely cluster number based on the ΔK criterion (Fig. 2). Cluster 1 comprised individuals from Giza and Minya; Cluster 2 comprised individuals from Dayrout and North Sinai (Fig. 3). However, this bipartitioning showed no contiguous or geographically meaningful spatial relationship, and the progressive decline in log-likelihood with increasing K indicated that K = 1 (panmixia) remained the most parsimonious interpretation of the data.

**Fig. 4:**
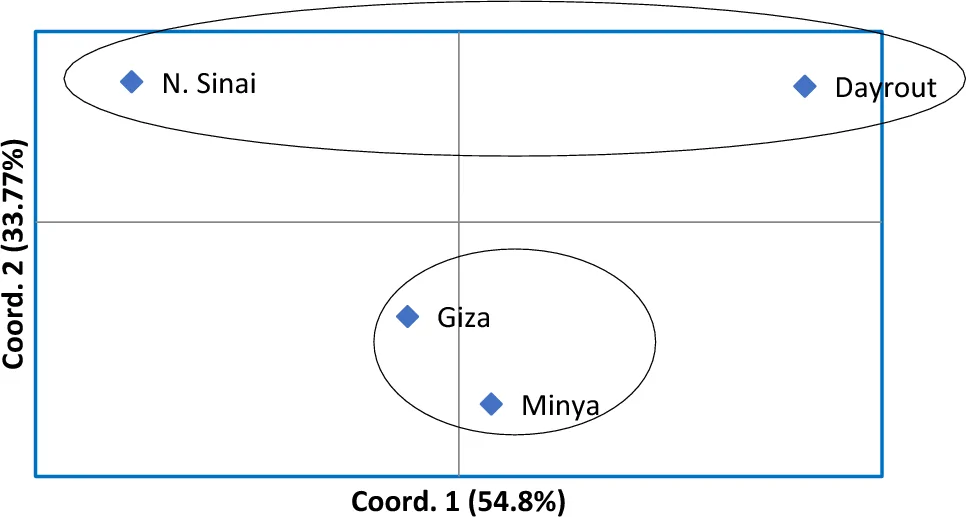
Principal coordinate analysis (PCoA) of *Chrysomya albiceps*based on pairwise ΦPT genetic distances. Coordinate 1 and Coordinate 2 explain 54.8% and 33.77% of total genetic variance, respectively. Two clusters are identified: Cluster 1 (Giza, Minya) and Cluster 2 (North Sinai, Dayrout).

Principal coordinate analysis (PCoA) on the pairwise Φ_PT_ distance matrix was congruent with the STRUCTURE outcome: two clusters emerged, Cluster 1 (Giza + Minya) and Cluster 2 (North Sinai + Dayrout), with the first two coordinates jointly accounting for 88.57% of total genetic variance (Coordinate 1: 54.8%; Coordinate 2: 33.77%) (Fig. 4). The absence of geographic coherence in both multivariate analyses corroborated the AMOVA inference of no isolation by distance.

#### Within-sample relatedness

**Fig. 5:**
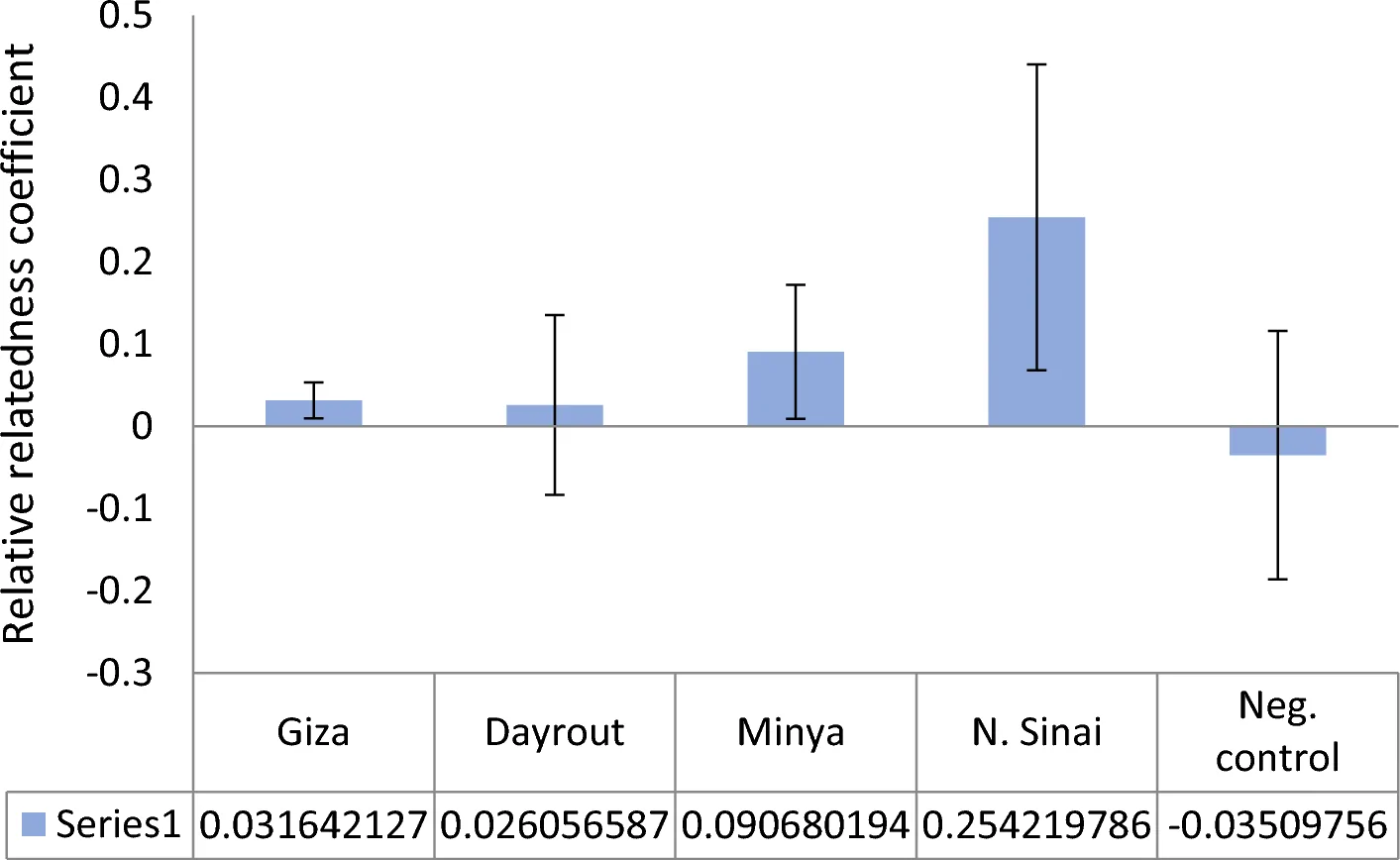
Mean pairwise relative relatedness coefficients (r ± SD) for *Chrysomya albiceps*within each population sample (Giza, Minya, Dayrout, North Sinai) and for the negative control (random individual per region). Positive values indicate within-sample kinship structure above random expectation.

Pairwise relative relatedness coefficients (r) estimated by SPAGeDi across the full *C. albiceps* dataset revealed consistently positive mean values in all four populations (overall mean r = 0.016 ± 0.009; range 0.02–0.25), indicating a within-sample excess of allele sharing relative to random expectations (Fig. 5). Nevertheless, absolute relatedness values were substantially lower than those recorded for *C. megacephala* (r = 0.321 ± 0.068) from the same study. The negative control (one individual selected at random from each region) yielded uniformly negative pairwise r values, confirming that the positive within-sample r values reflect genuine kinship structure rather than a methodological artifact. The comparatively low overall relatedness in *C. albiceps* is attributed to the extended collection period (3 h) and the pooling of individuals from two Dayrout sub-sites.

#### Population assignment analysis

Population assignment was performed on all 77 *C. albiceps* individuals using AFLPOP v2.0 at a minimum likelihood difference (MLD) threshold of 1. Under the *Simulation* procedure (1,000 random MCMC genotypes per population; 10 iterations), 3,999 of 4,000 simulated genotypes were correctly allocated to their source population (99.98% allocation success), with a single genotype from the Minya pool remaining unassigned (Table 3; Figure S2).

**Table 3:** AFLPOP Simulation results for *Chrysomya albiceps* (MLD = 1).

Allocated to →	Dayrout	North Sinai	Giza	Minya
Dayrout	1000	–	–	–
North Sinai	–	1000	–	–
Giza	–	–	1000	–
Minya	–	–	–	999
Unassigned	0	0	0	1

Mean number of correctly allocated simulated genotypes (out of 1,000 per population) across four source populations.

Under the *Re-allocation* procedure applied to empirical individuals, 52 of 77 individuals (67.53%) were correctly assigned to their population of origin, 18 (23.37%) were misallocated, and 7 (9.09%) remained unassigned (Table 4; Figure S3). The Dayrout population yielded the highest mis-allocation rate: of 24 Dayrout individuals, 9 were incorrectly allocated to Giza and 2 to Minya, with 1 unassigned. The discrepancy between *Simulation* (98.70%) and *Re-allocation* (67.53%) success rates was 31.17 percentage points, indicating that the observed *C. albiceps* genetic structure is insufficient to support reliable individual-level population assignment and therefore limits the utility of this species for inferring postmortem corpse relocation.

**Table 4:** AFLPOP leave-one-out re-allocation results for 77 *Chrysomya albiceps* individuals at a minimum likelihood difference (MLD) threshold of 1.

Assigned to	Dayrout (n = 24)	North Sinai (n = 8)	Giza (n = 36)	Minya (n = 9)
Dayrout	17	0	9	2
North Sinai	0	6	0	0
Giza	2	0	24	0
Minya	4	0	1	5
Unassigned	1	2	2	2

Columns indicate the population from which each individual was sampled, whereas rows indicate the population to which the individual was assigned by AFLPOP. Diagonal values represent correctly re-assigned individuals, off-diagonal values represent incorrect assignments, and the final row indicates individuals that could not be assigned to any population under the selected MLD threshold.

### Chrysomya marginalis

#### Specimen collection, AFLP reproducibility, and genotyping

A total of 27 adult *C. marginalis* specimens were collected from three temporally and/or spatially distinct sampling events: Giza 2013 (n = 7), Dayrout 2013 (n = 3), and Giza 2014 (n = 17) (Table 5). The Giza 2014 cohort was collected using a sweep net in an open fish market, whereas the 2013 collections were obtained from fish-baited traps.

**Table 5:** *Chrysomya marginalis* collection sites, sample sizes, collection dates, geographic coordinates, percentage of polymorphic AFLP loci, and within-sample Nei’s gene diversity (Hj).

Collection site	Coordinates	Collection date	n (sex)	% Polymorphism*	Hj ± SE
Giza 2013	30°00′N / 31°10′E	27 May 2013	7 (♀)	74.3	0.289 ± 0.006
Dayrout 2013	27°34′N / 30°49′E	4 June 2013	3 (♀)	74.7	0.320 ± 0.008
Giza 2014	30°00′N / 31°10′E	5 May 2014	17 (3♂, 14♀)	75.0	0.299 ± 0.003
Total	–	–	27	–	–

*Percentage of loci with minor allele frequency ≥ 5% of total AFLP loci scored.

AFLP reproducibility was assessed using 10 independent replicate profiles generated from a single *C. marginalis* DNA digestion–ligation product. Eight replicates were fully concordant across all 507 scored loci, whereas the remaining two each lacked a small number of alleles, corresponding to an overall error rate of 3.15%. This value is comparable to previously reported AFLP error rates for *Chrysomya* species, supporting the reliability of the genotyping protocol.

The four selective primer combinations generated 823 AFLP loci, of which 507 polymorphic loci were retained after filtering. All individuals exhibited unique multilocus genotypes. The proportion of polymorphic loci ranged from 74.3% to 75.0%, while Nei’s gene diversity (Hj) ranged from 0.289 to 0.320 across populations (Table 5).

#### Genetic and geographic structure

**Fig. 6:**
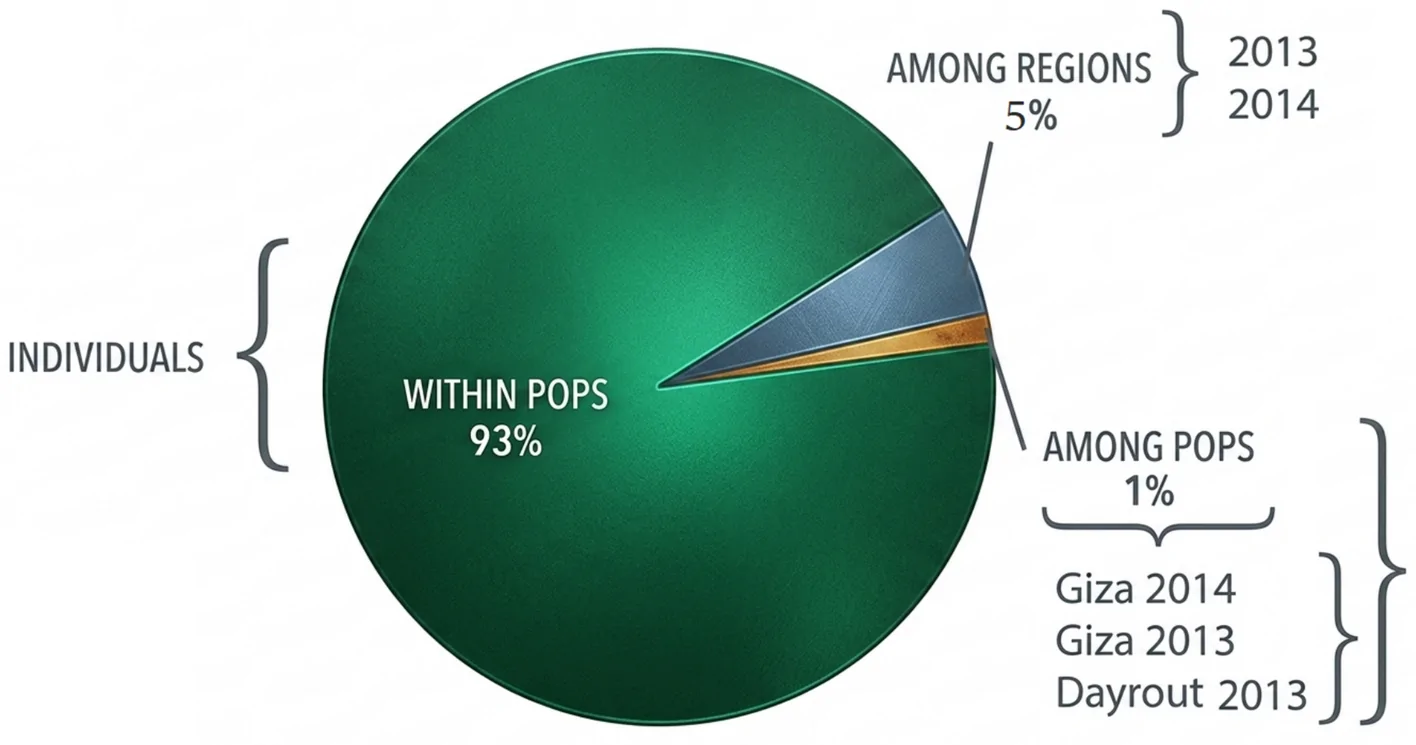
AMOVA-based partitioning of molecular variance in *Chrysomya marginalis*. The pie chart shows the proportional distribution of total genetic variation across hierarchical levels, with 5% attributed to among-region variation, 1% to among-population variation, and 93% residing within populations.

Three-level AMOVA (among regions, among populations within regions, within populations) partitioned genetic variation as follows: 5% among regions (ΦRT = 0.054, P = 0.078), 1% among populations within regions (ΦPR = 0.068, P = 0.020), and 93% within populations (Table 6; Fig. 6). The among-region component represented variation between the predefined temporal sampling groups (2013: Giza + Dayrout vs. 2014: Giza) and was not statistically significant (P = 0.078), indicating no robust evidence of temporal genetic differentiation at this hierarchical level. The largest proportion of variation occurred within populations (93%), with only minor differentiation observed among populations within regions (1%). Overall, the results indicate weak hierarchical genetic structure in *C. marginalis*, with genetic variation largely distributed at the within-population level.

**Table 6:** AMOVA results for *Chrysomya marginalis*. Based on 507 AFLP loci and 999 permutations. Regions are defined temporally (2013 collections: Giza + Dayrout vs. 2014 Giza).

Source	df	SS	MS	Est. Var	%*	Φ statistic	P
Among Regions	1	191.728	191.728	5.944	5%	ΦRT = 0.054	0.078
Among Pops (within Regions)	1	108.433	108.433	1.468	1%	ΦPR = 0.068	0.020
Within Populations	24	2454.431	102.268	102.268	93%	(not applicable)	(not tested)
Total	26	2754.593	–	109.680	100%	–	–

ΦPT is not shown here because there are only two hierarchical levels above individuals.

*Percentages are rounded to the nearest whole number; the sum may not equal exactly 100%.

The Mantel test between geographic and genetic (Φ_PT_) distances produced a non-significant strong positive correlation (R^2^ = 0.75, P = 0.340; SSx = 50,080.149, SSy = 0.000, SPxy = 4.294, Rxy = 0.872), indicating that geographic distance does not predict genetic differentiation in *C. marginalis* (Table S4; Figure S4). Correspondingly, F_st_ = 0.0318 ± 0.00 and total gene diversity H_T_ = 0.312 confirmed statistically significant but very slight genetic differentiation among sites (P = 0.001), without restriction of gene flow across the sampled spatial scale.

**Fig. 7:**
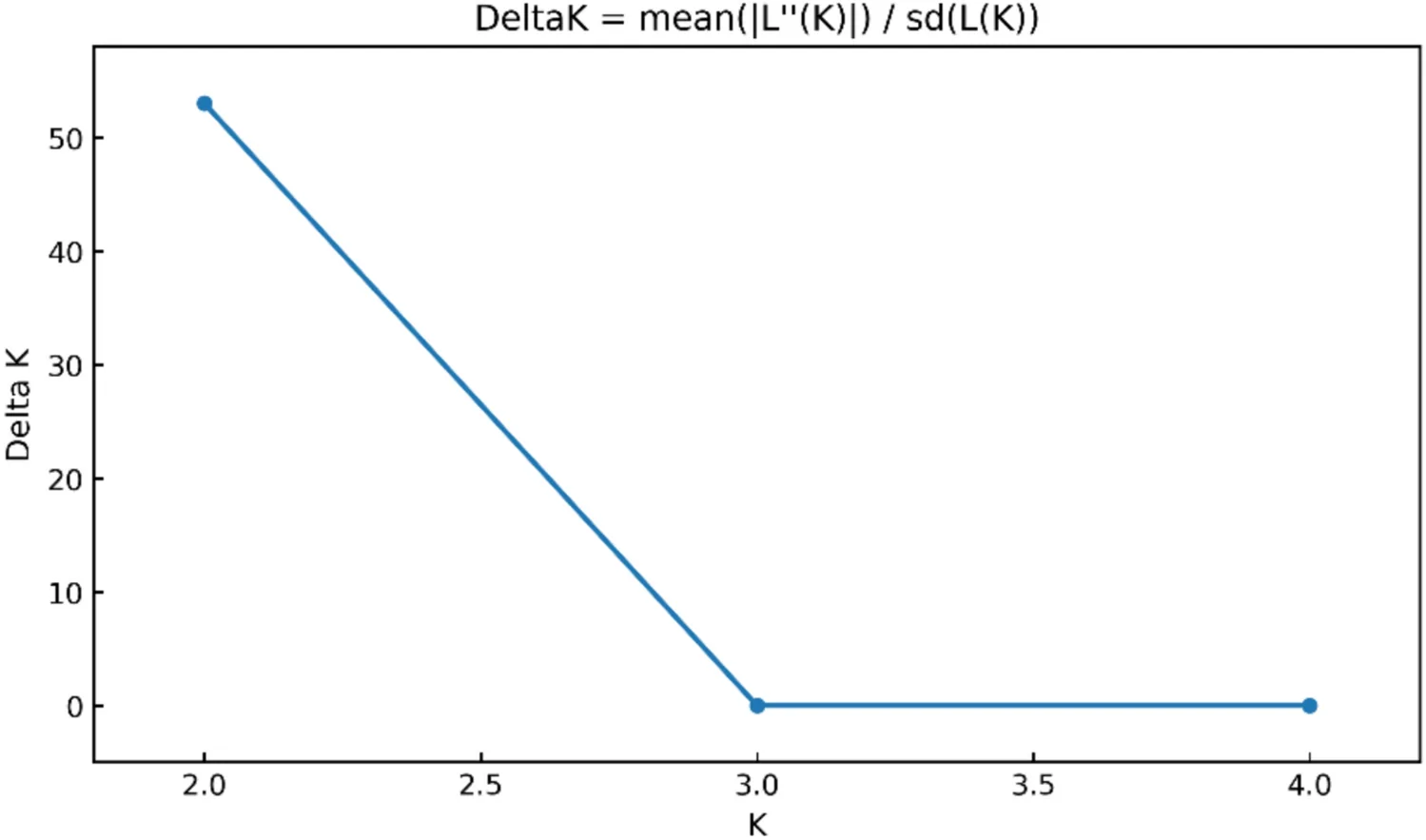
ΔK plot from STRUCTURE HARVESTER analysis of *Chrysomya marginalis*(K = 1–9), indicating maximum support at K = 2.

**Fig. 8:**
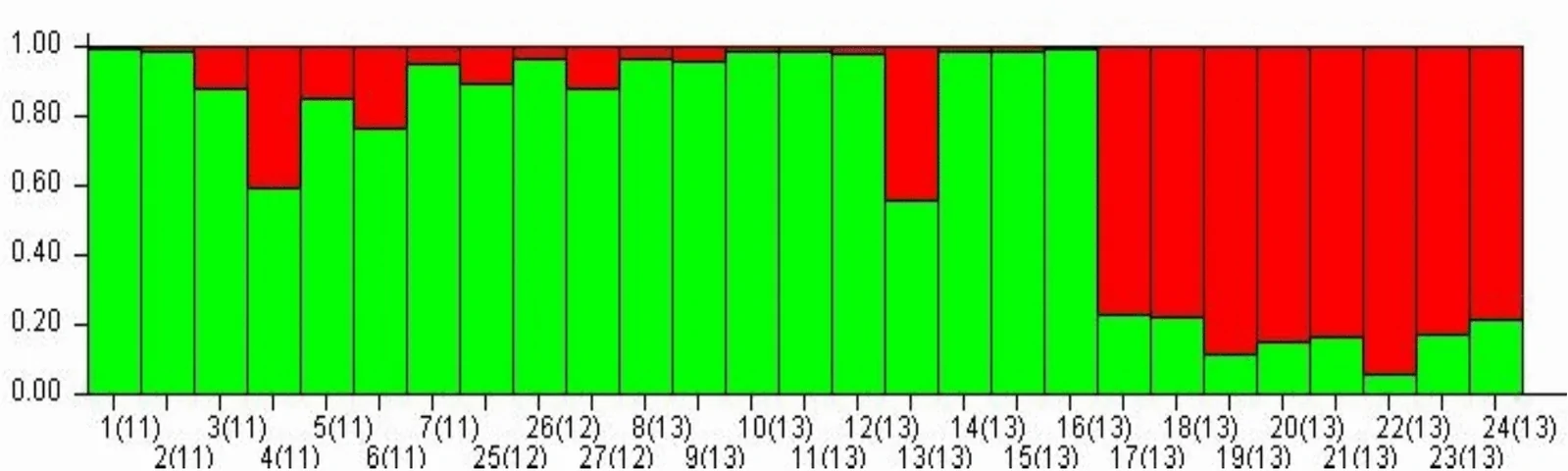
STRUCTURE bar plot for *Chrysomya marginalis* at K = 2. Each vertical bar represents one individual; colors denote estimated cluster membership proportions. Population codes: 11 = Giza 2013, 12 = Dayrout 2013, 13 = Giza 2014.

**Fig. 9:**
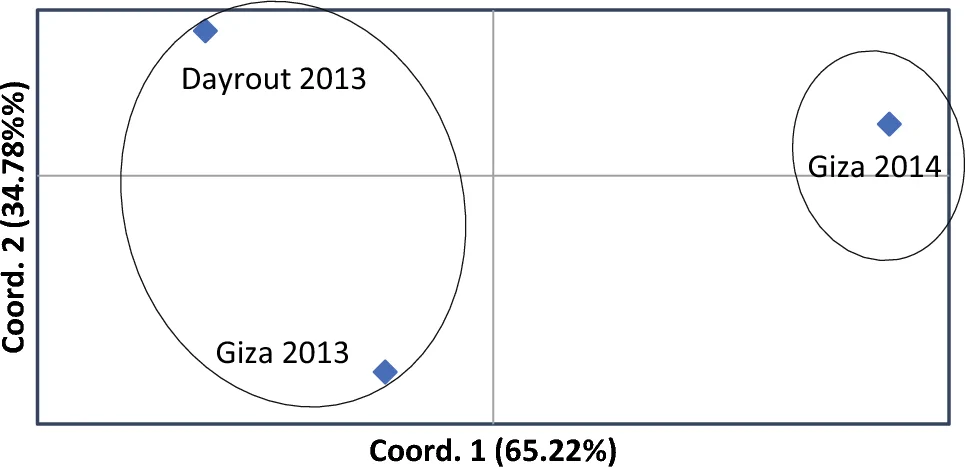
Principal coordinate analysis (PCoA) of *Chrysomya marginalis* based on pairwise ΦPT genetic distances. Coordinate 1 and Coordinate 2 explain 34.78% and 65.22% of total genetic variance, respectively. Cluster 1 = Giza 2013 + Dayrout 2013; Cluster 2 = Giza 2014.

Bayesian STRUCTURE analysis at K = 1–9 identified K = 2 as the most probable cluster solution based on the ΔK method (Fig. 7). Cluster 1 incorporated all 2013 individuals (Giza 2013 and Dayrout 2013), and Cluster 2 comprised the Giza 2014 collection (Fig. 8), revealing that temporal—rather than geographic—factors drove the detected bipartite structure. PCoA on pairwise Φ_PT_ distances was fully concordant with the STRUCTURE result, with the first two principal coordinates collectively explaining 100% of total genetic variation (Coordinate 1: 65.22%, Coordinate 2: 34.78%; Fig. 9). Cluster 1 (2013 samples) and Cluster 2 (Giza 2014) were fully resolved along Coordinate 1.

#### Within-sample relatedness

**Fig. 10:**
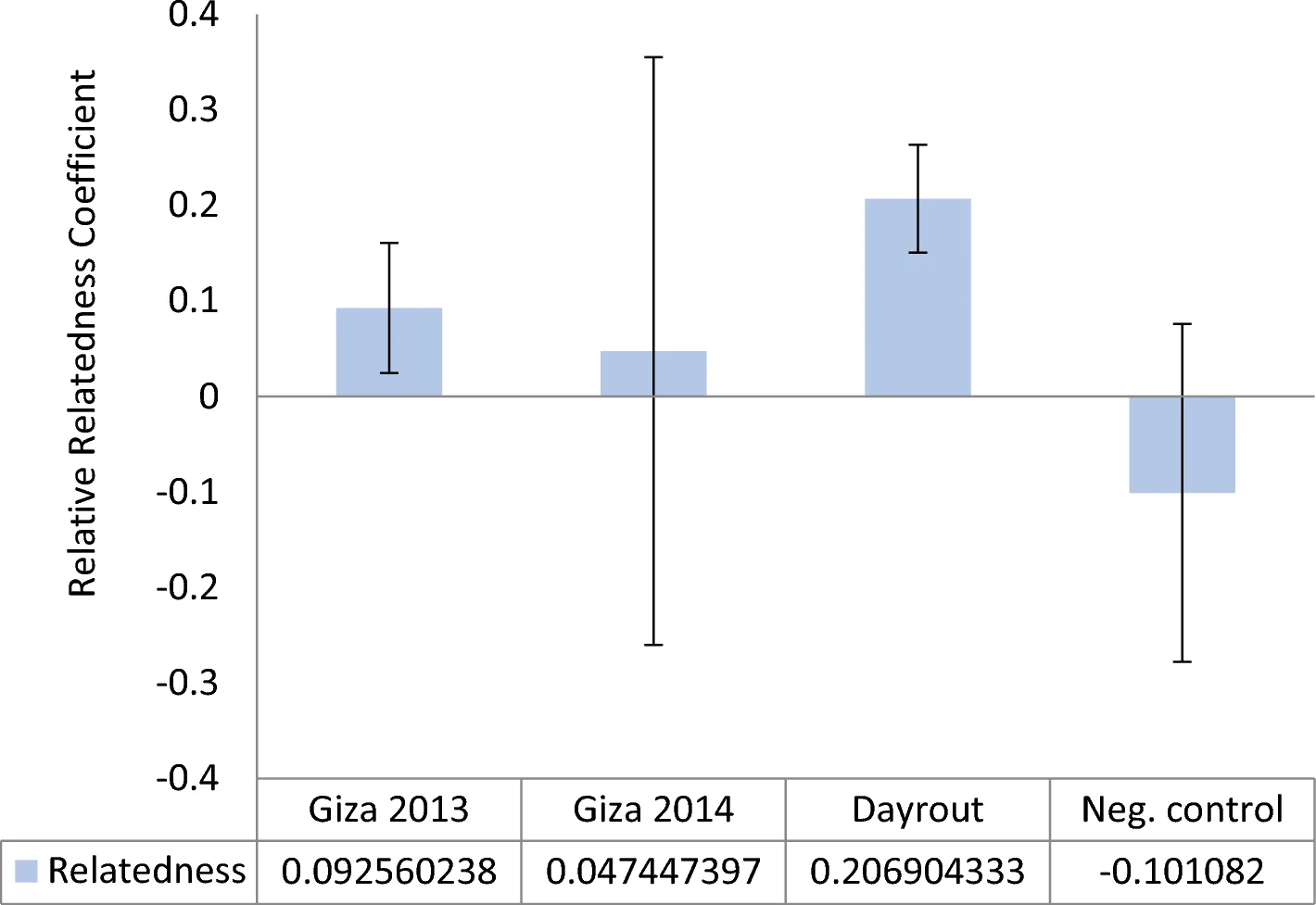
Mean pairwise relative relatedness coefficients (r ± SD) for *Chrysomya marginalis* within each population (Giza 2013, Dayrout 2013, Giza 2014) and for the negative control. Positive values denote within-sample kinship above random expectation.

Pairwise relative relatedness coefficients (r) for *C. marginalis* were uniformly positive across all three populations (overall mean r = 0.081 ± 0.008; range 0.04–0.20), indicating higher-than-random allele sharing within sampling events (Fig. 10). The negative control yielded uniformly negative pairwise r values. The mean relatedness of *C. marginalis* (r = 0.081 ± 0.008) was intermediate between that of *C. albiceps* (r = 0.016 ± 0.009) and *C. megacephala* (r = 0.321 ± 0.068), with the broader standard deviation likely reflecting the extended 3-h collection window and consequent inclusion of unrelated foragers.

#### Population assignment analysis

Assignment analysis was conducted on all 27 *C. marginalis* individuals using AFLPOP v2.0. The MLD threshold was optimized to 13 (i.e., a genotype was required to be 10^13^ times more likely in its putative source population than in any alternative) to minimize erroneous non-assignments. Under the *Simulation* procedure (1,000 random genotypes per population), 2,999 of 3,000 simulated genotypes were correctly allocated (99.97% simulation success), with one Giza 2014 genotype left unassigned (Table 7; Figure S5).

**Table 7:** AFLPOP Simulation results for *Chrysomya marginalis* (MLD = 13). Mean number of correctly allocated simulated genotypes (of 1,000 per population) across three source populations.

Allocated to →	Dayrout 2013	Giza 2013	Giza 2014
Dayrout 2013	1000	–	–
Giza 2013	–	1000	–
Giza 2014	–	–	999
Unassigned	0	0	1

Under the *Re-allocation* procedure, only 12 of 27 empirical individuals (44.44%) were correctly assigned to their source population; 2 individuals (7.40%) were misallocated to Giza 2013, and 13 individuals (48.14%) remained unassigned (Table 8; Figure S6). Notably, all 3 Dayrout 2013 individuals were unassigned, and 6 of 17 Giza 2014 individuals were also unassigned. Due to the very small sample size (n = 3), the Dayrout 2013 population provides an unreliable estimate of allele frequencies and was therefore excluded from interpretation of re-allocation success rates. Accordingly, the overall re-allocation success rate (44.44%) should be interpreted with caution, as it is influenced by this undersampled population. The gap between *Simulation* (96.29%) and *Re-allocation* (44.44%) success rates was 51.85 percentage points—the largest discrepancy observed among the three *Chrysomya* species examined—indicating that *C. marginalis* AFLP profiles are insufficiently discriminant for reliable individual assignment to source populations at the stringency level required for forensic application.

**Table 8:** AFLPOP re-allocation results for 27 *Chrysomya marginalis* individuals (MLD = 13).

Assigned to	Dayrout 2013 (n = 3)	Giza 2013 (n = 7)	Giza 2014 (n = 17)
Dayrout 2013	0	0	0
Giza 2013	0	3	2
Giza 2014	0	0	9
Unassigned	3	4	6

Columns represent the true (sampled) source populations, and rows represent the populations to which individuals were assigned by AFLPOP. Diagonal values indicate correctly assigned individuals, off-diagonal values indicate misassignments, and “Unassigned” indicates individuals that could not be assigned at the selected MLD threshold.

### Comparative summary of assignment performance across species

Table 9 consolidates the assignment analysis outcomes for all three Egyptian *Chrysomya* species examined in this study. Across species, *Simulation* success rates were uniformly high (96.29–99.97%), whereas *Re-allocation* success rates were substantially lower and species-specific (44.44–67.53%), reflecting the degree of realized population genetic structure. The simulation-to-reallocation discrepancy was smallest for *C. albiceps* (31.17 pp) and largest for *C. marginalis* (51.85 pp), with *C. megacephala* occupying an intermediate position. Among the three species, only *C. megacephala* met the criteria for forensically reliable individual assignment, based on its comparatively high F_st_ (0.158 ± 0.422), high H_T_ (0.207), and the narrowest simulation-reallocation discrepancy. Both *C. albiceps* and *C. marginalis* demonstrated insufficient genetic differentiation for unambiguous source-population assignment at the individual level.

**Table 9:** Comparative Summary of AFLP Loci, genetic diversity parameters, and assignment analysis outcomes for three Egyptian *Chrysomya* species. Discrepancy = Simulation success rate − Re-allocation success rate (pp = percentage points).

Parameter	*C* *albiceps* (Simulation)	*C. albiceps* (Re-alloc.)	*C. marginalis* (Simulation)	*C. marginalis* (Re-alloc.)	C. megacephala* (Simulation)	C. megacephala* (Re-alloc.)	Discrepancy
Polymorphic loci retained	542	–	507	–	590	–	–
Fst (± SD)	0.0453 ± 0.23	–	0.0318 ± 0.00	–	0.158 ± 0.422	–	–
HT	0.207	–	0.312	–	0.207	–	–
Mean r (± SE)	0.016 ± 0.009	–	0.081 ± 0.008	–	0.321 ± 0.068	–	–
MLD threshold	1	–	13	–	7*	–	–
Assignment success (%)	98.70	67.53	96.29	44.44	–*	–*	–*
Discrepancy (pp)	31.17	–	51.85	–	8.16*	–	–

*C. *megacephala* assignment data reported from Salem et al.^15^. MLD = minimum likelihood difference; HT = total gene diversity; Fst = fixation index; r = relative relatedness coefficient; pp = percentage points.

## Discussion

This study provides the first AFLP-based population genetic assessment of *C. albiceps* and *C. marginalis* in Egypt, and it shows that both species are characterized by high within-population diversity, weak among-population differentiation, and limited forensic assignment power under the present sampling design. The broad pattern is consistent with substantial regional connectivity rather than strong geographic subdivision, yet it is not equivalent to complete panmixia. Instead, the data suggest a system in which frequent movement among resource patches maintains gene flow, while temporal turnover and sampling context introduce subtle but detectable structure. In practical terms, the study indicates that these flies can carry informative population-level signal, but that this signal is currently too weak and too context dependent to support routine individual-level source attribution in forensic applications.

The AFLP dataset itself was technically robust and provided sufficient resolution to detect small shifts in allele frequencies. After filtering, 507–542 polymorphic loci were retained, and every individual had a unique multilocus genotype. This marker density is substantial for non-model insects and gives confidence that the weak structure observed here is biological rather than methodological. Even so, the AMOVA results for both species placed most variance within populations, and the estimated Fst values remained low. In *C. albiceps*, the among-population component was small but significant, whereas in *C. marginalis* the spatial signal was even weaker. These values are best interpreted as evidence of high connectivity with limited differentiation, not as proof of homogeneous populations in the strictest sense. The comparison with Egyptian *C. megacephala*^15^ is useful in this respect because it shows that the genus spans a gradient of structure rather than a single population-genetic pattern.

The absence of isolation by distance in both species further supports this interpretation. Geographic separation across the sampled scale did not predict genetic differentiation, which is consistent with a metapopulation framework in which ephemeral carrion resources are colonized by dispersing adults moving among local patches^35^. Blow flies are capable of substantial movement, and field studies have shown that some calliphorids can disperse several kilometres per day^36–40^. Similar low spatial structure has also been reported for *Lucilia sericata*^41^, *Phormia regina*^42^, and invasive *Chrysomya megacephala* in Florida^17^. The present findings extend that general pattern to Egyptian *C. albiceps* and *C. marginalis*, but they also show that connectivity does not eliminate all structure. Rather, it appears to reduce spatial differentiation to a level that is detectable statistically but limited biologically.

A more informative distinction emerges when temporal rather than spatial sampling is considered. In *C. marginalis*, the 2013 and 2014 Giza collections separated into distinct groups in the clustering and ordination analyses, suggesting that year-to-year turnover contributed more to the observed structure than locality did. In *C. albiceps*, STRUCTURE identified K = 2, but the resulting clusters were not geographically coherent, and the overall pattern is more parsimoniously explained by weak, nonspatial structure than by discrete population subdivision. This is a familiar issue in low-Fst datasets, where the ΔK statistic may detect apparent clustering even when the biological meaning of those clusters is limited^29,30^. The results therefore support a cautious reading: temporal cohort composition appears to matter, but the scale and stability of that signal vary by species and sampling event. Similar temporal structuring has been described in *P. regina*^43^, as well as in unrelated taxa such as gastropods^44^ and Atlantic salmon^45^, and can be understood as the outcome of sequential recruitment from ephemeral habitats rather than as evidence of stable geographic isolation. In that sense, the Egyptian *Chrysomya* data fit a broader ecological model in which time can be a stronger axis of differentiation than distance.

The relatedness analyses add an important layer to this interpretation. Both species showed positive within-sample pairwise relatedness, and the negative control behaved as expected, indicating that the signal is biological rather than analytical. The magnitude of relatedness was modest in *C. albiceps* and somewhat higher in *C. marginalis*, but both were much lower than the values previously reported for Egyptian *C. megacephala*^15^. That comparison suggests that sampling design and resource context may strongly shape the degree of kin aggregation captured in a collection event. Longer collections at open fish markets likely admitted a more heterogeneous mix of foraging adults, while shorter, more localized bait collections may have sampled fewer family groups. The present data do not directly test the underlying behavioral mechanism, so it would be too strong to attribute the kinship pattern to any single process. Still, the results are consistent with the long-recognized tendency of carrion flies to arrive and develop in cohorts rather than as random representatives of a regional gene pool^4,5,17,42,46,47^. That has practical consequences: flies collected from the same bait or maggot mass should not be assumed to be independent observations, and relatedness-based analyses may therefore complement, rather than replace, population assignment approaches under some forensic scenarios^5,46^.

The assignment analyses make the limits of the current datasets especially clear. Simulation-based allocation was near perfect for both species, showing that the marker sets contain enough information under idealized assumptions. Empirical re-allocation was much lower, however, and the gap between the two measures was substantial in both species, especially in *C. marginalis*. This disparity is exactly what would be expected when among-population differentiation is weak: the genotype space looks highly separable in silico, but real individuals fall into overlapping frequency distributions once finite sampling and low structure are taken into account^16^. For *C. albiceps*, the misallocation rate was nontrivial even at low stringency, and for *C. marginalis* nearly half of the specimens remained unassigned despite the highly stringent likelihood threshold. The small Dayrout sample in *C. marginalis* also likely reduced the stability of allele-frequency estimates and contributed to high non-assignment, which is an important practical limitation. In aggregate, these results indicate that the current AFLP profiles are not yet reliable for routine individual-level source attribution in either species. The comparison with Egyptian *C. megacephala*^15^ is again instructive, because it shows that assignment performance improves when differentiation is stronger, but also that even moderate structure does not guarantee forensic usefulness unless the sampling framework is sufficiently representative.

The broader comparative context reinforces this conclusion. Among the Egyptian *Chrysomya* species examined so far, *C. megacephala* appears to occupy the upper end of the observed differentiation gradient, with higher Fst, stronger kin structure, and better assignment performance^15^. *C. albiceps* and *C. marginalis* fall on the lower end of that spectrum. This species-level variation matters because it shows that forensic applicability cannot be inferred from genus membership or from marker density alone; it must be established empirically for each taxon and region. Similar caution is warranted from studies of other blow flies. Low spatial structure in *L. sericata*^41^, *P. regina*^42^, and *Lucilia mexicana*^47^ demonstrates that cosmopolitan carrion flies often share a high-connectivity population history, but the degree of detectable differentiation still differs across species and study systems. Recent work on phenotypic polymorphism in the genus *Chrysomya* also underscores the need for species-level resolution when interpreting heterogeneity in morphology or genetics^48^. The present study therefore contributes less by identifying a universal pattern than by showing that even closely related forensic flies can differ meaningfully in how much population structure they retain.

These results also have implications for laboratory colony establishment and the interpretation of developmental reference data. Colonies founded from a single collection event may capture only a small subset of the standing genetic variation in a natural population, especially if the source sample is kin-structured. That concern has already been raised for *P. regina*^49^ and is relevant here as well. Although the relatedness values in *C. albiceps* and *C. marginalis* were lower than those in *C. megacephala*^15^, the positive within-sample signal indicates that single-event collections are not guaranteed to represent the full allelic diversity of the regional pool. This matters because laboratory rearing histories can shape developmental measurements that are later used for postmortem interval estimation^9–12^. The practical implication is not that the present species are unsuitable for developmental work, but that colonies should be founded from multiple independent sampling events spread across time and space whenever possible, so that reference data are not inadvertently anchored to a narrow set of related founders^49^.

An important consideration in interpreting these results is the heterogeneous sampling design, including differences in collection method, sampling duration, and sample size, all of which may have influenced relatedness estimates, genetic clustering, and assignment accuracy. In *C. albiceps*, the 3-h collection period and pooling of individuals from two nearby Dayrout sites may have reduced apparent relatedness by increasing the likelihood of sampling unrelated foraging adults. In *C. marginalis*, the very small Dayrout 2013 sample (n = 3) likely reduced the precision of allele-frequency estimates, potentially affecting STRUCTURE clustering and contributing to the high proportion of unassigned individuals. Accordingly, the observed patterns should be interpreted with these sampling limitations in mind, and future studies should employ standardized collection protocols and more balanced sampling designs.

Several additional limitations should be noted. The study examined adults rather than larvae from known carcass cases; therefore, the findings should be interpreted cautiously when extrapolating to forensic casework. Also, nearly all specimens analyzed were females, with only three males included in the *C. marginalis* Giza 2014 population and no males in the *C. albiceps* dataset. Because AFLP markers are autosomal and the present study did not investigate sex-specific genetic structure or dispersal, the small number of male specimens is unlikely to have materially influenced estimates of genetic diversity, relatedness, or population assignment. Nevertheless, future studies incorporating more balanced male-to-female ratios would be valuable for assessing whether sex-biased dispersal or demographic differences contribute to population genetic structure in Egyptian *Chrysomya* populations.

Although AFLP provided sufficient genome-wide coverage to detect broad patterns of genetic differentiation and relatedness in the present study, future investigations employing high-density SNP markers or reduced-representation sequencing approaches (e.g., RAD-seq or GBS), together with larger, more balanced samples and larval collections from known forensic cases, are expected to provide greater power for resolving fine-scale population structure, improving forensic assignment accuracy, and clarifying the biological basis of the patterns observed here^50–56^.

In summary, Egyptian *C. albiceps* and *C. marginalis* appear to be highly connected but only weakly structured populations in which temporal turnover and kin-structured collection events contribute more to detectable differentiation than geography does. The AFLP data are strong enough to reveal this pattern, but not strong enough to support reliable individual-level source assignment under the present sampling conditions. That conclusion is important because it sets a realistic boundary on what these species can contribute to forensic inference. Rather than offering a universal solution, the data show that forensic utility in carrion flies is species specific, context dependent, and tightly constrained by the amount of population differentiation actually present. For *C. albiceps* and *C. marginalis*, the present evidence supports cautious use, broader sampling, and further marker development before routine forensic assignment can be considered robust.

## Conclusions

This study provides a high-resolution population genetic analysis of Egyptian *C. albiceps* and *C. marginalis*. Despite extensive polymorphic loci, both species exhibited low genetic differentiation and weak spatial structure, reflecting high dispersal capacity and extensive gene flow. The observed patterns are more consistently explained by temporal turnover and cohort dynamics than by geographic separation. Positive within-sample relatedness indicates that field collections are inherently kin-structured, arising from clustered oviposition and synchronized development. This kin aggregation, together with temporal variability, explains the presence of weak but detectable genetic structure despite overall connectivity. From a forensic perspective, the low differentiation in *C. albiceps* and *C. marginalis* limits the reliability of population assignment for source attribution. In contrast, Egyptian *C. megacephala*^15^ exhibits moderate differentiation and correspondingly higher assignment power, highlighting that forensic applicability is strongly dependent on population structure. Overall, *C. albiceps* and *C. marginalis* in Egypt represent highly connected, weakly structured systems shaped by gene flow, kin structure, and temporal dynamics. Enhancing their forensic utility will require increased sampling depth, broader geographic coverage, and incorporation of temporal replication to improve resolution of population differentiation.

## Supplementary Information


Supplementary Information.


## Data Availability

Data supporting this study are included within the article and/or its supplementary materials.

## Abbreviations

AFLP: Amplified fragment length polymorphism
AMOVA: Analysis of molecular variance
BSA: Bovine serum albumin
CE: Capillary electrophoresis
df: Degrees of freedom
DNA: Deoxyribonucleic acid
F_ST_: Fixation index
FAM: Fluorescein amidite (fluorescent dye)
GBS: Genotyping-by-sequencing
Hj: Nei’s gene diversity within a population
HT: Total gene diversity across all populations
IBD: Isolation by distance
LIZ: Internal size standard (Applied Biosystems)
MCMC: Markov chain Monte Carlo
MLD: Minimum likelihood difference
MS: Mean square
mtDNA: Mitochondrial DNA
NFW: Nuclease-free water
%P: Percentage of polymorphic loci (minor allele frequency ≥ 5%)
PCR: Polymerase chain reaction
PCoA: Principal coordinate analysis
PMI: Postmortem interval
ΦPR: Proportion of genetic variance among populations within regions
ΦPT: Proportion of genetic variance among populations relative to total
ΦRT: Proportion of genetic variance among regions
r: Pairwise relative relatedness coefficient
RADseq: Restriction site-associated DNA sequencing
RAPD: Random amplified polymorphic DNA
RFU: Relative fluorescence units
SD: Standard deviation
SE: Standard error
SNP: Single nucleotide polymorphism
SPAGeDi: Spatial pattern analysis of genetic diversity (software)
SPSS: Statistical Package for Social Sciences
SS: Sum of squares
STR: Short tandem repeat
