# Supplementary material for: Gene flow, kinship, and source population assignment in Chrysomya blow flies
Source: Sci Rep. 2026 Aug 29;16:27111. doi: 10.1038/s41598-026-66124-w (PMC13526017; doi:10.1038/s41598-026-66124-w)
Supplement: Supplementary file 1 [file 41598_2026_66124_MOESM1_ESM.docx]

**Gene Flow, Kinship, and Source Population Assignment in *Chrysomya* Blow Flies**

**SUPPLEMENTARY MATERIALS**

## Supplementary Tables

**Table S1. Collection sites, sampling dates, geographic coordinates, and sample sizes for *Chrysomya* *albiceps* and *C*. *marginalis* used in AFLP population genetic analysis.**

| ***Species*** | **Collection Site** | **Year** | **Coordinates** | **Collection Date** | **n (sex)** |
| --- | --- | --- | --- | --- | --- |
| *C. albiceps* | Giza | 2013 | 30°00′N, 31°10′E | 27 May 2013 | 36 (♀) |
|  | Minya | 2013 | 30°07′N, 30°33′E | 13 March 2013 | 9 (♀) |
|  | Dayrout | 2013 | 27°34′N, 30°49′E | 4 June 2013 | 24 (♀) |
|  | North Sinai | 2013 | 31°02′N, 33°00′E | 2 May 2013 | 8 (♀) |
| *C. marginalis* | Giza | 2013 | 30°00′N, 31°10′E | 27 May 2013 | 7 (♀) |
|  | Dayrout | 2013 | 27°34′N, 30°49′E | 4 June 2013 | 3 (♀) |
|  | Giza | 2014 | 30°00′N, 31°10′E | 5 May 2014 | 17 (3♂, 14♀) |
| **Total** | — | — | — | — | **104** |

n = number of individuals included in analysis. ♀ = female; ♂ = male. Dayrout 2013 *C*. *albiceps* sample includes individuals from a supplementary site < 10 km from the primary trap location (see text).

**Table S2. Sequences of AFLP adaptors and selective primers used in this study. All oligonucleotides were synthesised by Integrated DNA Technologies (Coralville, IA, USA).**

| **Name** | **Sequence (5′ → 3′)** | **Role** |
| --- | --- | --- |
| *EcoRI adaptor 1* | CTCGTAGACTGCGTACC | Adaptor annealing to EcoRI-cut ends |
| EcoRI adaptor 2 | AATTGGTACGCAGTCTAC | Adaptor annealing to EcoRI-cut ends |
| *PstI adaptor 1* | CTCGTAGACTGCGTACATGCA | Adaptor annealing to PstI-cut ends |
| PstI adaptor 2 | TGTACGCAGTCTACG | Adaptor annealing to PstI-cut ends |
| *EcoRI+A (pre-selective)* | GACTGCGTACCAATTCA | Pre-selective amplification |
| PstI+A (pre-selective) | GACTGCGTACATGCAGA | Pre-selective amplification |
| *PstI+ACG (selective)* | GACTGCGTACATGCAGACG | Selective amplification |
| PstI+AGT (selective) | GACTGCGTACATGCAGAGT | Selective amplification |
| *PstI+AAC (selective)* | GACTGCGTACATGCAGAAC | Selective amplification |
| PstI+ATC (selective) | GACTGCGTACATGCAGACAT | Selective amplification |

Selective PstI primers were each paired individually with the FAM-labelled EcoRI primer in separate selective PCR reactions, yielding four primer combinations per individual.

**Table S3.** Mantel test statistics for *Chrysomya albiceps*: correlation between geographic (km) and genetic (Φ_PT_) distance matrices based on 999 permutations.

| **SSx** | **SSy** | **SPxy** | **Rxy** | **P(rxy-rand ≥ rxy-data)** |
| --- | --- | --- | --- | --- |
| 60,384.408 | 0.0181 | −12.879 | −0.388 | 0.326 |

**Table S4.** Mantel test statistics for *Chrysomya marginalis*: correlation between geographic (km) and genetic (Φ_PT_) distance matrices based on 999 permutations.

| **SSx** | **SSy** | **SPxy** | **Rxy** | **P(rxy-rand ≥ rxy-data)** |
| --- | --- | --- | --- | --- |
| 50,080.149 | 0.000 | 4.294 | 0.872 | 0.340 |

## Supplementary Figures

GGD vs PhiPTP

Genetic distance

| Geographic distance (Km) | Y |
| --- | --- |
| 274.081 | 0.0428904 |
| 213.805 | 0.0542917 |
| 440.071 | 0.0338009 |
| 107.089 | 0.143537 |
| 282.786 | 0.0815253 |
| 309.9 | 0.182541 |

**Figure S1.** Mantel test scatter plot: geographic distance (km) versus genetic distance (Φ_PT_) for *Chrysomya albiceps*. Each point represents a pairwise population comparison.

|  | Dayrout | N.Sinai | Giza | Minya | None |
| --- | --- | --- | --- | --- | --- |
| Dayrout | 1000 | 0 | 0 | 0 | 0 |
| N.Sinai | 0 | 1000 | 0 | 0 | 0 |
| Giza | 0 | 0 | 1000 | 0 | 0 |
| Minya | 0 | 0 | 0 | 999.9 | 0.1 |

**Figure S2.** AFLPOP *Simulation* allocation matrix for *Chrysomya albiceps* (MLD = 1). Bars represent the proportion of 1,000 simulated genotypes per source population correctly assigned to each putative destination.

|  | Dayrout | N.Sinai | Giza | Minya | None |
| --- | --- | --- | --- | --- | --- |
| Dayrout | 17 | 0 | 2 | 4 | 1 |
| N.Sinai | 0 | 6 | 0 | 0 | 2 |
| Giza | 9 | 0 | 24 | 1 | 2 |
| Minya | 2 | 0 | 0 | 5 | 2 |

**Figure S3.** AFLPOP *Re-allocation* allocation matrix for *Chrysomya albiceps* (MLD = 1). Bars represent the proportion of 77 empirical individuals assigned to each putative destination population.

GGD vs PhiPTP

Genetic distance

| Geographic distance (Km) | Y |
| --- | --- |
| 0 | 0.0433858 |
| 274.081 | 0.0592583 |
| 274.081 | 0.0745135 |

**Figure S4.** Mantel test scatter plot: geographic distance (km) versus genetic distance (Φ_PT_) for *Chrysomya marginalis*.

|  | Dayrout 2013 | Giza 2013 | Giza 2014 | None |
| --- | --- | --- | --- | --- |
| Dayrout 2013 | 1000 | 0 | 0 | 0 |
| Giza 2013 | 0 | 1000 | 0 | 0 |
| Giza 2014 | 0 | 0 | 999.9 | 0.1 |

**Figure S5.** AFLPOP *Simulation* allocation matrix for *Chrysomya marginalis* (MLD = 13). Bars represent the proportion of 1,000 simulated genotypes per source population correctly assigned to each putative destination.

|  | Dayrout 2013 | Giza 2013 | Giza 2014 | None |
| --- | --- | --- | --- | --- |
| Dayrout 2013 | 0 | 0 | 0 | 3 |
| Giza 2013 | 0 | 3 | 0 | 4 |
| Giza 2014 | 0 | 2 | 9 | 6 |

**Figure S6.** AFLPOP *Re-allocation* allocation matrix for *Chrysomya marginalis* (MLD = 13). Bars represent the proportion of 27 empirical individuals assigned to each putative destination population.
